# Development of a novel elastomer with unique properties as fire and radiation resistance

**DOI:** 10.1038/s41598-024-72190-9

**Published:** 2024-09-27

**Authors:** Tarek Mansour Mohamed, Ghada A. Mahmoud

**Affiliations:** Polymer Chemistry Department, National Center for Radiation Research and Technology (NCRRT), Egyptian Atomic Energy Authority (EAEA), Cairo, Egypt

**Keywords:** Carboxymethyl cellulose, Acrylic acid, Gamma radiation, Elastomer, Radiation resistance, Chemistry, Materials science

## Abstract

This study aims to create a novel, distinct form of elastomer with superior ability to resist fire, high resistance to radiation, and resistance to environmental conditions such as temperature and solvents. This type of natural-based elastomer was prepared using carboxymethyl cellulose CMC, polyacrylic acid PAA, crosslinked with tannic acid TA. Most techniques in elastomer manufacture technologies are unfriendly and participate in increasing carbon emissions. Gamma radiation was used as a clean tool for copolymerization and crosslinking the elastomer. The irradiation dose of 5 kGy with a rate of 3.32 kGy/h was enough to produce CMC/PAA/TA elastomer. The properties of the produced elastomer were investigated by Fourier-transformed infrared spectroscopy (FT-IR), X-ray diffraction, thermal gravimetric analysis (TGA), and limiting oxygen index (LOI). CMC/PAA/TA has high resistance to solvents such as acetone, benzene, HCl, and HNO_3_. The tensile strength is 3.376 MPa, the elongation percent is 501.689%, and the LOI value is 30%. The produced elastomer possessed excellent gamma radiation resistance. The elastomer was exposed later to 1864 kGy of gamma radiation without showing degradation and retained its properties, as confirmed by FTIR, TGA, and mechanical properties. After investigation, it can be inferred that the produced CMC/PAA/TA elastomer exhibited outstanding properties.

## Introduction

Elastomer is a type of polymer that exhibits elastic properties; it can deform under stress and revert to its initial form once the load is removed^1^. Generally it has weak Young’s modulus and elevated failure strain relative to other materials^2^. Elastomers have unique molecular structures that give them their distinctive properties. They are composed of long polymer chains interconnected through various types of cross-linking bonds^3^. The strong cross-linking hydrogen bonds confer robustness and elasticity^4^. When stressed, these cross-links allow the chains to move and stretch out and then relax back to their original state once the stress is removed. Elastomers are often referred to as rubber materials due to their rubbery consistency and flexibility^5^. They find applications in various industries, including automotive^6^, aerospace^7^, construction^8^, electronics^9^, and healthcare^10,11^. They are used in the manufacturing of tires^12^, seals^13^, gaskets^14^, hoses^15^, vibration dampers^15^, O-rings^16^, medical gloves^17^, and many other products that require elasticity and resilience. The common examples of elastomers include natural rubber (made from the latex of rubber trees), synthetic rubbers like styrene-butadiene rubber (SBR), neoprene, and silicone rubber. Natural rubber is derived from a sustainable and renewable resource; it is biodegradable, it can break down naturally over time without causing long-lasting environmental harm^18^. However, it has relatively lower mechanical strength compared to some synthetic materials^19^. This limitation may affect its suitability for certain industrial applications that require high mechanical properties or specific performance requirements. Synthetic rubber offers a versatile and customizable alternative to natural rubber, allowing for the production of materials with tailored properties to meet specific industrial needs^20^. The extraction and processing of these non-renewable resources contribute to carbon emissions and environmental deterioration. Additionally, the disposal of synthetic rubber waste can pose challenges, as it may not readily degrade or be recyclable in some cases. Silicone rubber is made from silicon, oxygen, carbon, and hydrogen atoms. It is a polymer formed through the cross-linking of silicone-based polymers, which results in a rubbery material with unique properties^21^. While silicone rubber offers many advantages, it also has some disadvantages. Silicone rubber is more expensive than other types of synthetic rubbers or elastomers. The production process and raw material costs contribute to its higher price.

Carboxymethyl cellulose CMC is an anionic polymer with carboxyl groups (–COOH) generated by an alkali-catalyzed cellulose reaction^22^. In aqueous solutions, CMC undergoes ionization, releasing carboxylate ions –COO^−^ and hydrogen ions H^+^. CMC is sensitive to pH variations. CMC remains coiled or aggregated under low pH levels (acidic environments), resulting in lower solubility and viscosity. CMC deprotonates as the pH rises (in alkaline conditions). Polyacrylic acid PAA is also an anionic polymer contains repeating carboxylic acid groups (–COOH) along its polymer chain^23^. PAA is also pH-responsive; at low pH (acidic conditions), PAA remains in its neutral form. As the pH increases (alkaline conditions), the carboxylic acid groups become ionized, resulting in increased solubility and swelling of the polymer. Tannic acid TA is a large polyphenolic compound with a complex structure^24,25^. It is composed of glucose molecules esterified with gallic acid, forming a polymer chain. It has multiple hydroxyl (–OH) groups and aromatic rings. These hydroxyl groups contribute to its antioxidant activity and reactivity.

With considering the overall environmental impact, reduced carbon emissions, and biodegradability, new elastomer was constructed from carboxymethyl cellulose/polyacrylic acid (CMC/PAA) and tannic acid TA is used as a cross-linker. A huge number of hydrogen bonds was performed to obtain the suitable elastic properties. The blended mixture was irradiated by gamma radiation inducing free radicals polymerization and crosslinking as a clean technique. various types of cross-linking bonds for desirable properties were obtained. The characteristics of the obtained natural-based elastomer were investigated.

## Material and methods

### Materials

Carboxymethyl cellulose 99%, tannic Acid 99%, nitric acid 53–55%, and acetone 99.5% were obtained from PIOCHEM for laboratory chemicals (Egypt). Acrylic acid 98.05% and sulphuric acid 97% were provided from ADVENT CHEMBIOPVT. LTD (India). Other solvents were obtained as follow: ethanol 99.9%, Brand chemicals (Egypt), Dimethyl formamide (DMF), El nasr pharmaceutical chemicals Co. (Egypt), benzene 100% annular, VWR chemicals (France), and hydrochloric acid (HCl) 30–32%, SPHINX (Egypt).

### Preparation of CMC/PAA/TA elastomer

Some pre-experiments were done on different formulations of CMC: AAc to determine which the best was. Two grams of CMC were completely disintegrated in 70 mL of deionized water with stirring at 70 °C to complete dissolving. 20 mL of acrylic acid was included after down to room temperature, and it was stirred for 20 min for dissolution. Followed by adding 0.5 g of tannic acid that was prior dissolved in hot water. The solution was completed to 100 mL with continuous stirring until a complete homogeneity. The solution mixture was poured into a glass Petri dish and exposed to gamma radiation at a dose of 5 kGy with a rate of 3.32 kGy/h. After irradiation, the obtained film was washed with tap water to exclude the un reacted materials and non-crosslinked ones, and finally, the crosslinked elastomer film (100% crosslinked) was dried in an oven at 50 °C.

### Solvent resistance

A sample of known mass (W_0_) was immersed in specific solvent and every day reweight for two weeks (W_1_) after that the surface solvent was dried with a filter paper. The soluble fraction was obtained using the following equation:

1 $${\text{Soluble fraction}}\;\left( \% \right) = \left( {\frac{{W_{0} - W_{1} }}{{W_{0} }}} \right) \times 100$$

### FT-IR analysis

The FT-IR was performed with a Bruker Unicom infrared spectrophotometer (Germany) within the400–4000 cm^−1^ wavelength range.

### TGA analysis

The TGA was done by Shimadzu TGA-30 (Japan) in a nitrogen environment from 30 to 600 °C at 10 °C/min heating rate.

### Mechanical properties

Tensile investigations were carried out using Hounsfield tensile testing equipment, (model H10 KS) on dumbbell-shaped specimens with a 50 mm length and 4 mm neck width at ambient. The speed of film stretched was 10 mm/min and using a 20 kN load cell.

### XRD analysis

Shimadzu Diffractometer D6000 series Kyoto, Japan was used for the XRD analysis. (30 mA and 40 kV) at Cu Kα (λ = 1.54 Å) radiation at ambient temperature with a 2–90 scan speed of 8 degrees per minute.

### Limiting oxygen index (LOI)

FTA-LOI made by Rheometeric Scientific Ltd, England, was used to measure LOI in accordance with ISO-4589. 100 × 20 × 3 mm^3^ is the sample size.

## Results and discussion

**Fig. 1:**
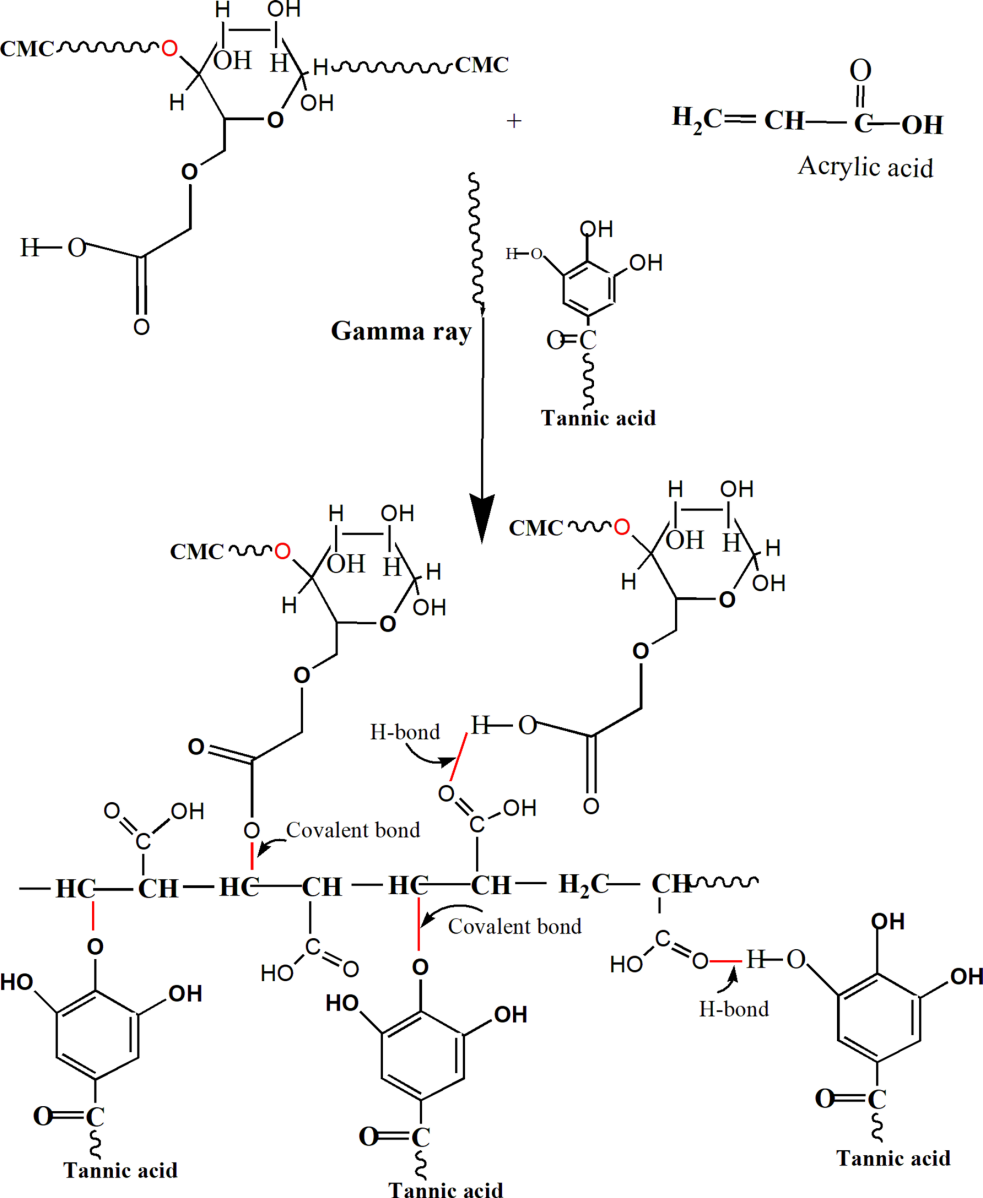
The possible reaction mechanism for CMC/PAA/TA elastomer.

**Fig. 2:**
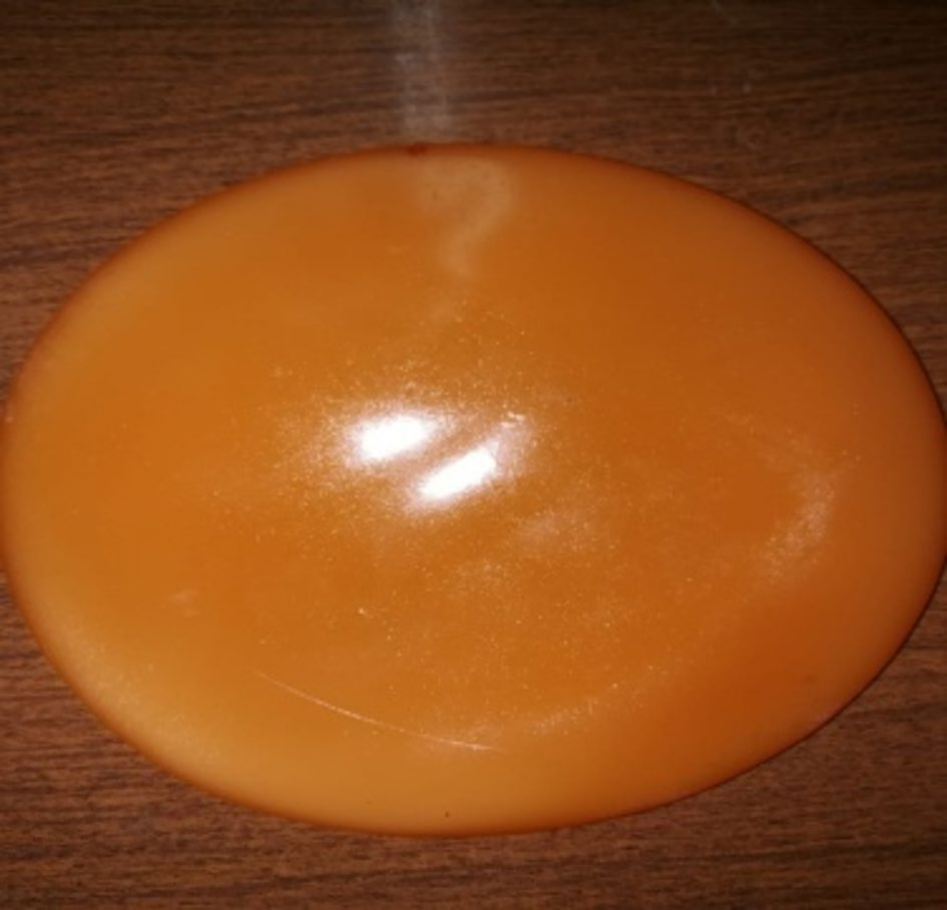
photo image of CMC/PAA/TA elastomer.

The objective of this research was to prepare elastomer has multiple properties that can be used in different applications. The purpose of this research was to create an elastomer with diverse qualities that can be employed in a variety of applications. At the same time the elastomer is based on environmentally friendly, low-cost materials. It is critical to use a sustainable preparation technique free of harmful chemicals and additives to achieve a product with high purity and no contaminants. The properties of elastomer created from the cross-linking chemical bonds and flexibility from the stretched hydrogen bonds. When mixing CMC with acrylic acid AA monomer hydrogen bonds was created between them (Fig. 1 first step). The addition of TA in the reaction medium as a cross-linker creates more hydrogen bonds as seen in Fig. 1 (second step). When CMC, AA monomer, and TA mixture solution was irradiated by gamma radiation, Fig. 1 (second step). The exposure of this ingredient to radiation AA can undergo polymerization under the influence of gamma radiation by formation of free radicals as the result of cleavage of π bonds and formed polyacrylic acid chain (PAA), also, the radiolysis of gamma radiation can form active species as active radicals on CMC and tannic acid which react with other radicals of PAA chain, this process can lead to the formation of crosslinked networks between the CMC, TA molecules and PAA polymer chains. This can result in the formation of a three-dimensional structure frame work with potentially altered properties compared to the original CMC and AA. At the same time AA can generate ester bonds by reacting with –OH on the cellulose and tannic acid backbone. The final product is shown in digital photo Fig. 2.

### Solvent resistance

The solvent resistance of elastomers^26^, or the capacity of elastomeric materials to endure exposure to various solvents, is of great importance in many industries and applications. Elastomers are commonly used in applications where they come into contact with different chemicals, such as in seals, gaskets, O-rings, and hoses. Solvents can have varying chemical compositions and properties, and some solvents may cause swelling, degradation, or other adverse reactions in elastomers. Solvent resistance ensures that the elastomer remains chemically compatible, allows for longer service life, and reduces the need for frequent replacements or repairs. By selecting the appropriate solvent-resistant elastomer for a specific application, industries can enhance reliability, efficiency, and safety in their operations.

Table 1 shows the soluble fraction (%) of CMC/PAA/TA in different solvents. As seen in Table 1, CMC/PAA/TA elastomer has high resistance to acidic and alkaline solvents such as acetone, benzene, HCl, sodium hydroxide and DMF. About 5.45% was soluble in HNO_3_ and 14% was soluble in ethanol and about 12% was soluble in case of sodium hydroxide after soaking for 2 weeks. It clear that from the obtained data, the prepared elastomer possesses high resistivity to many deferent type of solvents in case of acidic and alkaline medium as shown from the table.

**Table 1:** Soluble fraction (%) after two weeks of CMC/PAA/TA in different solvents.

Solvents	Weight_dry_ (g)	Weight_dry after soaking_ (g)	Soluble fraction (%)
Acetone	1.17	1.19	~ 0
Benzene	1.08	1.07	0.92
Nitric acid	1.1	1.04	5.45
Hydrochloric acid	1.07	1.05	1.86
*N*,*N*-dimethylformamide	1.21	1.22	~ 0
Ethanol	1.07	0.92	14
Sodium hydroxide (pH = 13.6)	2.26	2.01	12%

### XRD analysis

**Fig. 3:**
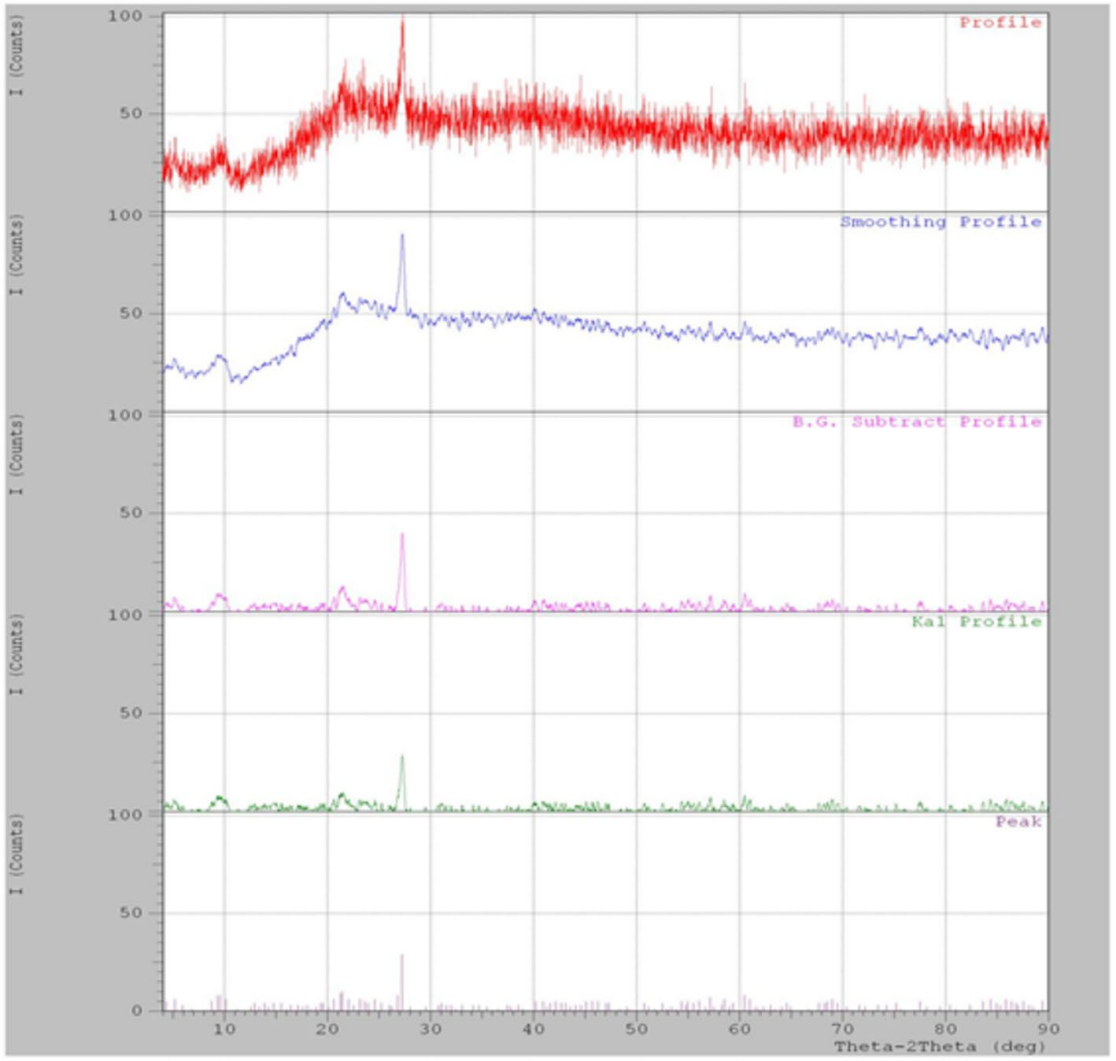
XRD patterns of CMC/PAA/TA.

Figure 3 depicts the XRD pattern of CMC/PAA/TA. The diffractogram reflected the amorphous structure of the prepared CMC/PAA/TA elastomer has a broad peak at 2θ = 22.0°. A diffraction peak at 28.0°, which confirmed some crystallinity in the polymeric structure that may be due to the arrangement on the crystal domain.

### Flame retardant of the prepared elastomer

Limiting oxygen index LOI is the lowest oxygen percentage in an oxygen–nitrogen mixture that is just enough to enable combustion of the specimen following ignition, indicating the capacity of materials to tolerate fire^27^. The higher the LOI values, the greater the capacity to withstand fire and the more difficult it is to ignite materials. The flammability properties of CMC/PAA/TA elastomer are examined by LOI. It was found that the elastomer has a LOI value of 30%. Because TA has a strong carbon-forming ability and anti-oxidant capacity, its presence improves the flame retardancy of CMC/PAA/TA elastomer^28^.

### FT-IR analysis

**Fig. 4:**
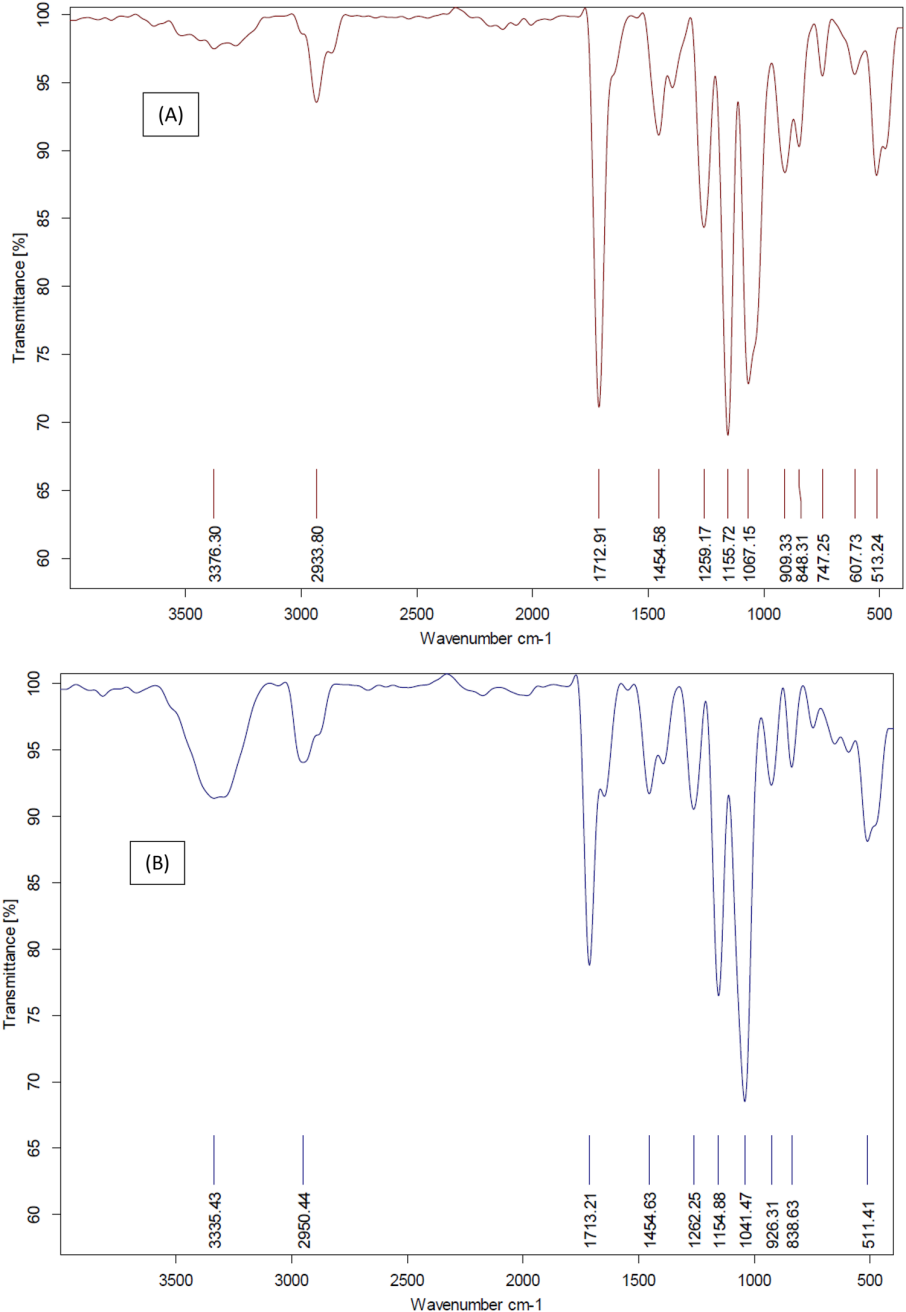
FTIR spectra of CMC/PAA/TA (**A**) before Gamma irradiation and (**B**) after exposure to1864 kGy gamma rays.

FTIR spectra of CMC/PAA/TA elastomer before and after exposure to 1864 kGy of gamma rays were investigated in Fig. 4. One of the prominent spectral characteristics found in the CMC/PAA/TA FTIR spectrum is a band centered at 3040 cm^−1^, due to O–H stretching vibrations of carboxylic group (Fig. 4A). The asymmetric stretching vibrations of C–H appears at 2931 cm^−1^^28,29^, which confirmed by the bending vibration band at 1042 cm^−1^. The band at 1735 cm^−1^ due to C=O stretching vibrations^30^. The splitting pattern of C=O may be related to the influence of hydrogen bonds. The band at 1138 cm^−1^ is caused by C–O stretching vibrations in ether linkages between aromatic rings in lignin. The intensity of the O–H and –CH bands increased while C=O decreased after exposure to 1864 kGy of gamma rays for CMC/PAA/TA (Fig. 4B), which may be due to a little degradation in the matrix^31^.

### Thermal properties of prepared elastomer

**Fig. 5:**
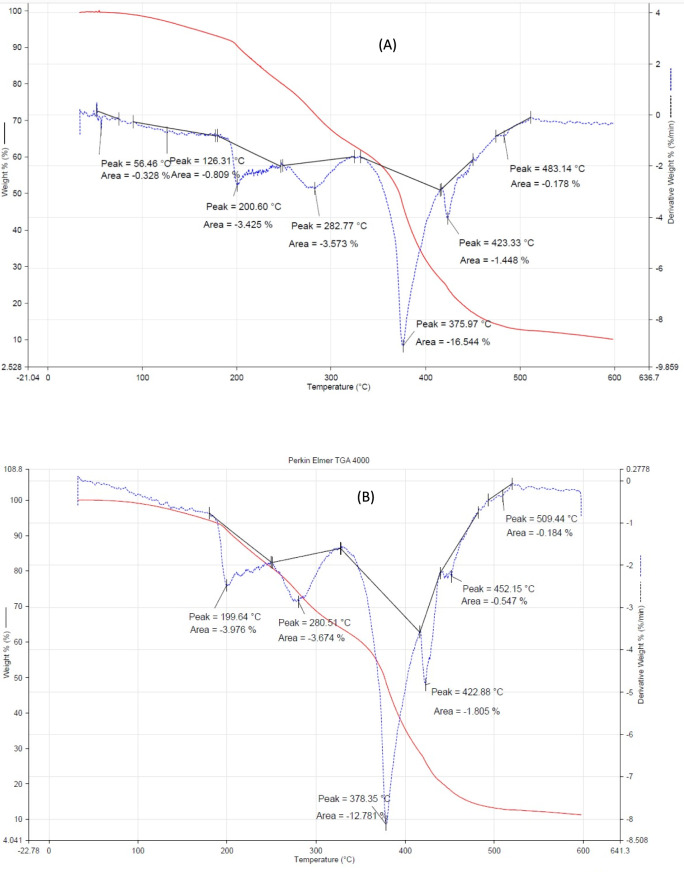
TGA of CMC/PAA/TA (**A**) before Gamma irradiation and (**B**) after exposure to1864 kGy gamma rays.

Figure 5 shows of CMC/PAA/TA before and after exposure to 1864 kGy of gamma rays. Under a nitrogen atmosphere, the specimens were tested at temperatures ranging from 30 to 600°°C at a constant rate of 20°°C/min. The TGA curves reveal that all specimens had three separate stages of weight reduction. The first stage of the thermogram of CMC/PAA/TA (Fig. 5A) shows the evaporation of partially physically bound water at 200.60 °C, whereas the second stage at 282.77 °C shows the disintegration of CMC and PAA side chains. The final stage, at 395.97 °C, reveals the disintegration of the polymer's primary chain. For CMC/PAA/TA after exposure to 1864 kGy of gamma rays (Fig. 5B), the same behavior was observed. A negligible change was observed that means the CMC/PAAc keep its thermal characteristic after exposure to gamma rays up to1864 kGy. The first stage was at 199.64 °C, the second stage at 280.75 °C, and the main decomposition stage at 378.35 °C. It must be noted that the investigation of the influence of gamma radiation was chosen up to 1864 kGy only as a limited example for the study.

### Mechanical properties

**Fig. 6:**
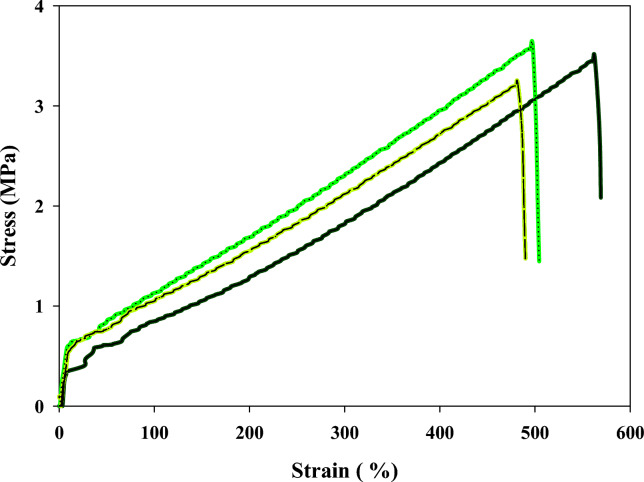
Stress/Strain curve of CMC/PAA/TA elastomer before irradiation.

The mechanical properties of elastomers are of significant importance due to their direct impact on the performance and functionality of elastomeric materials in various applications. Elastomers should possess sufficient strength to withstand the loads and forces they encounter during use. An elastomer's tensile stress is commonly defined by its tensile strength, which is the greatest stress or force that the material can sustain before failing or breaking^32^. Elastomers are known for their high tensile strength. Elastomers often face challenges such as tearing due to contact with rough surfaces, sharp edges, or repeated friction. Good tear resistance ensures that elastomers can withstand the application-specific forces without developing cracks or ruptures. Young’s modulus, also referred to as the modulus of elasticity, is an indicator of the stiffness or rigidity of a material. Young’s modulus is an essential mechanical property in materials science and engineering as it helps engineers and designers understand and predict how materials will respond to applied forces, Young modulus are calculated from stess/strain curve as shown in Table 4. Elongation percent indicates the maximum amount of deformation a material can undergo before it breaks or fails. It is a measure of the material's ductility or ability to be drawn into a wire-like shape without breaking. The stress/strain curve of CMC/PAA/TA was examined in Fig. 6, and Table 2 summarizes the data. CMC/PAA/TA displayed good mechanical properties,, as indicated in Table 2, where the tensile strength is ~ 3.376 MPa, Yong’s Modules is ~ 0.595 MPa, tear strength is 33.754 N/mm, and the elongation percent is ~ 501.689%. The mechanical properties of CMC/PAA/TA after exposure to gamma rays at an irradiation dose of 1864 kGy were investigated as obtained in Fig. 6 and the analyzed data was performed in Table 3. As explained in Table 3, the tensile strength is ~ 3.087 MPa, Young’s Modules is ~ 0.606 MPa, the tear strength is 30.87 N/mm, and the elongation percent is ~ 468.461%. The result is considered excellent, CMC/PAA/TA elastomer kept good mechanical properties after exposure to a high radiation dose, 1864 kGy. This means the CMC/PAA/TA elastomer resists the gamma radiation at least up to the studied value.

**Table 2:** Mechanical properties data of CMC/PAA/TA.

No.	Force @ peak (N)	Elong. @ peak (mm)	Elong. @ break (mm)	Tensile stress (MPa)	Yong's modules (MPa)
1	88.358	84.451	85.377	3.465	0.475
2	97.107	74.65	75.657	3.597	0.601
3	80.664	72.289	73.504	3.227	0.556
4	80.339	64.198	66.475	3.214	0.748
Maximum	97.107	84.451	85.377	3.597	0.748
Minimum	80.339	64.198	66.475	3.214	0.475
Mean	86.617	73.897	75.253	3.376	0.595

No.	Tear strength (N/mm)		Elongation percentage @ break (%)		
1	34.65		569.178		
2	35.966		504.383		
3	32.265		490.027		
4	32.136		443.167		
Maximum	35.966		569.178		
Minimum	32.136		443.167		
Mean	33.754		501.689

**Table 3:** Mechanical properties data of CMC/PAA/TA after exposure to1864 kGy of gamma rays.

No.	Force @ peak (N)	Elong. @ peak (mm)	Elong. @ break (mm)	Tensile stress (MPa)	Yong’s modules (MPa)
1	77.801	69.611	70.867	3.112	0.554
2	56.317	61.215	63.661	2.537	0.76
3	84.695	69.014	70.485	3.388	0.595
4	82.787	75.56	76.064	3.311	0.515
Maximum	84.695	75.56	76.064	3.388	0.76
Minimum	56.317	61.215	63.661	2.537	0.515
Mean	75.4	68.85	70.269	3.087	0.606

No.	Tear strength (N/mm)		Elongation percentage @ break (%)		
1	31.121		472.444		
2	25.368		424.407		
3	33.878		469.9		
4	33.115		507.091		
Maximum	33.878		507.091		
Minimum	25.368		424.407		
Mean	30.87		468.461		

#### Calculating of Young modulus data

**Fig. 7:**
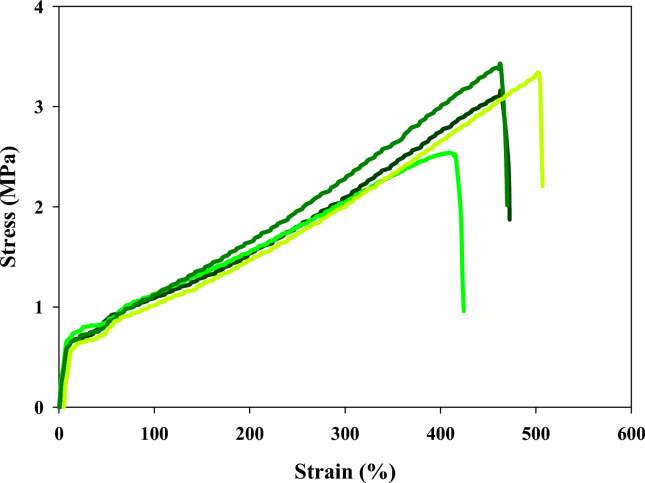
Stress/Strain curve of CMC/PAA/TA elastomer after irradiation with 1864 kGy.

Youngs modulus are used to determine the materials stiffens and can be calculated from stress–strain curve by dividing the tensile stress/tensile strain, here, according to obtained data from Figs. 6 and 7 the Youngs modulus is calculated as the following in Table 4.

**Table 4:** Calculated Youngs modulus from stress–strain curve of CMC/PAA/TA before and after exposure to 1864 kGy gamma rays.

No.	Young modules (MPa) Sample 1 before exposure to gamma radiation	Young modules (MPa) Sample 2 after exposure to gamma radiation
1	0.475	0.554
2	0.601	0.76
3	0.556	0.595
4	0.748	0.515
Mean	0.595	0.606

It can be noted that the resistance of CMC/PAA/TA elastomer to gamma radiation where it mainly keeps its properties through the investigated radiation dose, 1864 kGy. The reasons of this behavior may due to the chemical structure of the elastomer; the tightly bonded molecular structures tend to be more resistant to radiation. Also, the highly cross-linked structure makes the material more stable and less susceptible to radiation-induced degradation. The inclusion of TA in the matrix may enhance the radiation resistance due to stearic hindrance of catechol groups of its structure. TA is considered as antioxidant and radical scavenger^33^, which can scavenge free radicals generated by radiation, preventing them from causing chain scission or other forms of degradation. By comparing the obtained properties data of CMC/PAA/TA elastomer with acrylic rubber (ACM) properties^34–36^, it can be noted that CMC/PAA/TA superior properties, particularly chemical and physical strength, and flame resistance. Additionally, when compared with ACM elastomer lacks gamma radiation protection and fire retardant properties.

## Conclusions

Carboxymethyl cellulose/polyacrylic acid/tannic acid CMC/PAA/TA elastomer was prepared using gamma radiation technology. This formulation was produced an elastomer with superior properties. The properties of elastomer created from the cross-linking chemical bonds and flexibility from the stretched hydrogen bonds. It has higher resistance to solvents such as acetone, benzene, HCl, and DMF. About 5.45% was soluble in HNO_3_, and 14% was soluble in ethanol after soaking for two weeks. The elastomer has good thermal stability and mechanical properties. The presence of TA improves the flame retardancy of CMC/PAA/TA elastomer where LOI value is 30%. CMC/PAA/TA was found to be resist to gamma radiation. This property was examined by exposure to gamma rays up to 1864 kGy, no remarkable change in properties was obtained. This result was proved by FTIR, TGA, and the mechanical properties investigation.

## Data Availability

Data which support this study will be made available when request from corresponding author.
